# Asymptomatic ventricular dilatation precedes clinical decline in rodent adult chronic communicating hydrocephalus

**DOI:** 10.1186/2045-8118-12-S1-O13

**Published:** 2015-09-18

**Authors:** Ignacio Jusue-Torres, Jennifer Lu, Eric W Sankey, Tito Vivas-Buitrago, Joshua Crawford, Mikhail Pletnikov, Jiadi Xu, Ari Blitz, Barbara Crain, Alicia Hulbert, Hugo Guerrero-Cazares, Oscar Gonzalez-Perez, Alfredo Quiñones-Hinojosa, Pat McAllister, Daniele Rigamonti

**Affiliations:** 1 Johns Hopkins University. School of Medicine, Department of Neurosurgery, USA; 2 Johns Hopkins University, School of Medicine, Department of Psychiatry and Behavioral Sciences, USA; 3 Johns Hopkins University, School of Medicine, F. M. Kirby Research Center for Functional Brain Imaging at the Kennedy Krieger Institute, USA; 4 Johns Hopkins University, School of Medicine, Department of Radiology and Radiological Science, USA; 5 Johns Hopkins University, School of Medicine, Department of Pathology, Division of Neuropathology, USA; 6 Johns Hopkins University, School of Medicine, Department of Oncology, USA; 7 University of Colima , Facultad de Psicologia, Laboratory of Neuroscience, Mexico; 8 Washington University, School of Medicine in St Louis, Department of Neurosurgery, USA

## Introduction

The pathogenesis and behavioral effects of normal pressure hydrocephalus (NPH) are not fully understood, and the temporal relationship between radiological changes and neurological deterioration is unknown.

## Methods

Bilateral subarachnoid injections of kaolin were administered in the cranial convexities of 20 adult rats. MRI was obtained using a Bruker Biospec 11.7 T MRI scanner at 14, 60, 90 and 120 days post kaolin injection. Locomotor, gait, and cognitive studies were performed independently every 2 weeks by faculty blinded to the imaging results. Tests included open field test, gait analysis, rotarod and novel object recognition. Logistic regression analysis was performed to assess association between ventricular size and clinical deterioration and rate of ventricular size enlargement and clinical deterioration.

## Results

Radiological ventricular size showed progressive growth over time at all times (p<0.0001). The fastest ventricular enlargement happened within the first two months. No changes in gait, cognition, anxiety and general locomotor activity were detected during the first two months. The first gait deterioration occurred at 69 days; anxiety at 80 days; cognitive at 81 days and locomotor after 120 days. At the end of the study 66% of rats developed gait deterioration, 66% cognitive deterioration and 83% anxiety changes. Ventricular enlargement was not associated with gait (p>0.05), cognitive (p>0.05) or anxiety (p>0.05) deterioration. Locomotor deterioration was associated with ventricular size (p=0.014), speed of ventricular enlargement (p=0.015) and extension of injected kaolin (p=0.04).

## Conclusions

Kaolin injected in the subarachnoid space of adult rats can produce slow onset communicating hydrocephalus. Initially the ventricular enlargement seen on images is asymptomatic. Ventricular enlargement does not correlate with clinical impairment with exception of delayed locomotor impairment.
